# The senolytic cocktail, dasatinib and quercetin, impacts the chromatin structure of both young and senescent vascular smooth muscle cells

**DOI:** 10.1007/s11357-024-01504-6

**Published:** 2025-01-20

**Authors:** Agnieszka Gadecka, Natalia Nowak, Edyta Bulanda, Dorota Janiszewska, Magdalena Dudkowska, Ewa Sikora, Anna Bielak-Zmijewska

**Affiliations:** 1 Laboratory of Molecular Basis of Aging, Nencki Institute of Experimental Biology, Polish Academy of Sciences, 3 Pasteur St., 02-093 Warsaw, Poland; 2 Laboratory of Calcium Binding Proteins, Nencki Institute of Experimental Biology, Polish Academy of Sciences, 3 Pasteur St., 02-093 Warsaw, Poland; 3 Laboratory of Imaging Tissue Structure and Function, Nencki Institute of Experimental Biology of Polish Academy of Sciences, 3 Pasteur St., 02-093 Warsaw, Poland; 4 Faculty of Chemistry, Department of Biotechnology of Medicines and Cosmetics, Warsaw University of Technology, 3 Noakowskiego St., 00-664 Warsaw, Poland

**Keywords:** Senolytics, D + Q, Chromatin structure, VSMC, Senescence, Aging

## Abstract

One promising strategy to alleviate aging symptoms is the treatment with senolytics that is compounds which selectively eliminate senescent cells. Some therapies aim to reduce symptoms of cellular senescence without senescent cell eradication (senomorphic activity). However, senotherapies raise many questions concerning the selectivity, safety and efficiency of senolitic drugs. A vital question is how the senolytic compounds affect young proliferating cells. In our study, we checked the impact of quercetin and dasatinib (D + Q), one of the promising drug mixtures of drugs, on chromatin structure in young and senescent cells. We analyzed the effect of a single and triple drug treatment on vascular smooth muscle cells. We have shown that D + Q impacts the chromatin in both young and senescent cells. In senescent cells, D + Q caused some symptoms of chromatin “rejuvenation” but in young cells some changes characteristic of senescent cells were observed. The alterations in young cells appeared only transiently and chromatin returned to the initial state after 24 h of recovery. The complexity of chromatin staining and nucleus morphology evaluation indicated that a triple treatment makes senescent cells more similar to the young ones than a single treatment. However, the analysis of senescence markers suggested that a single treatment with D + Q caused slightly less pronounced senescence characteristics and was more efficient in alleviating the features of senescence than a triple treatment. It is still an open question whether the alterations caused by D + Q are beneficial or harmful in the long term; however, so far, it can be concluded that the effects depend on cell type and the physiological context.

**Supplementary Information:**

The online version contains supplementary material available at 10.1007/s11357-024-01504-6.

## Introduction

Ever since it has been proven that cellular senescence is directly responsible for age-related health problems, senescent cells have been recognized as a potential therapeutic target to alleviate the symptoms of aging [1, 2]. Eradication of senescent cells seems to be an excellent way to reduce their impact on the surrounding tissues and diminish low-grade inflammation, a characteristic feature of age-associated alterations [3]. One of the select and promising mixtures of drugs with senolytic activity consists of dasatinib and quercetin (D + Q) [4]. Dasatinib (tyrosine kinase inhibitor) is a drug that has been used for a long time in cancer therapy [5]. However, for senotherapeutic purposes, the drug's doses and administration frequency are lower [4]. Quercetin is a well-known natural flavonoid, widely used as a nutraceutical with many beneficial properties, such as antioxidative and anti-inflammatory activity. Its senolytic activity is related to inhibition of the PI3K and Bcl-2 family members [6]. Each drug has different cell specificity; dasatinib is senolytically active in preadipocytes, and quercetin in endothelial cells [4]. When administered together they are more effective and have a broader impact on more cell types [4]. The D + Q mixture was tested in animal natural aging and animal models of age-related diseases such as atherosclerosis, fibrotic pulmonary disease, type 2 diabetes and Alzheimer's disease [3]. We have shown that D + Q administration for 8 weeks alleviated aging-related cognitive impairments in old rats [7]. Promising animal studies prompted the use of this mixture in clinical trials [8]. The first human studies showed a reduced senescent cell burden in the adipose tissue of patients suffering from diabetic kidney disease [9] and improved physical functions in patients with idiopathic pulmonary fibrosis [10]. Several pilot trials for AD have been completed [11]. However, it remains unclear whether treatment with senolytics is safe for young cells, and what treatment schedule would be most effective. Moreover, cellular senescence is a fundamental cellular process involved in tissue regeneration, protection from fibrosis, and arrest of cancer cell proliferation [12]. Given the physiological role of cellular senescence it should be firmly established if removal of senescent cells can be ultimately beneficial.

**Fig. 1:**
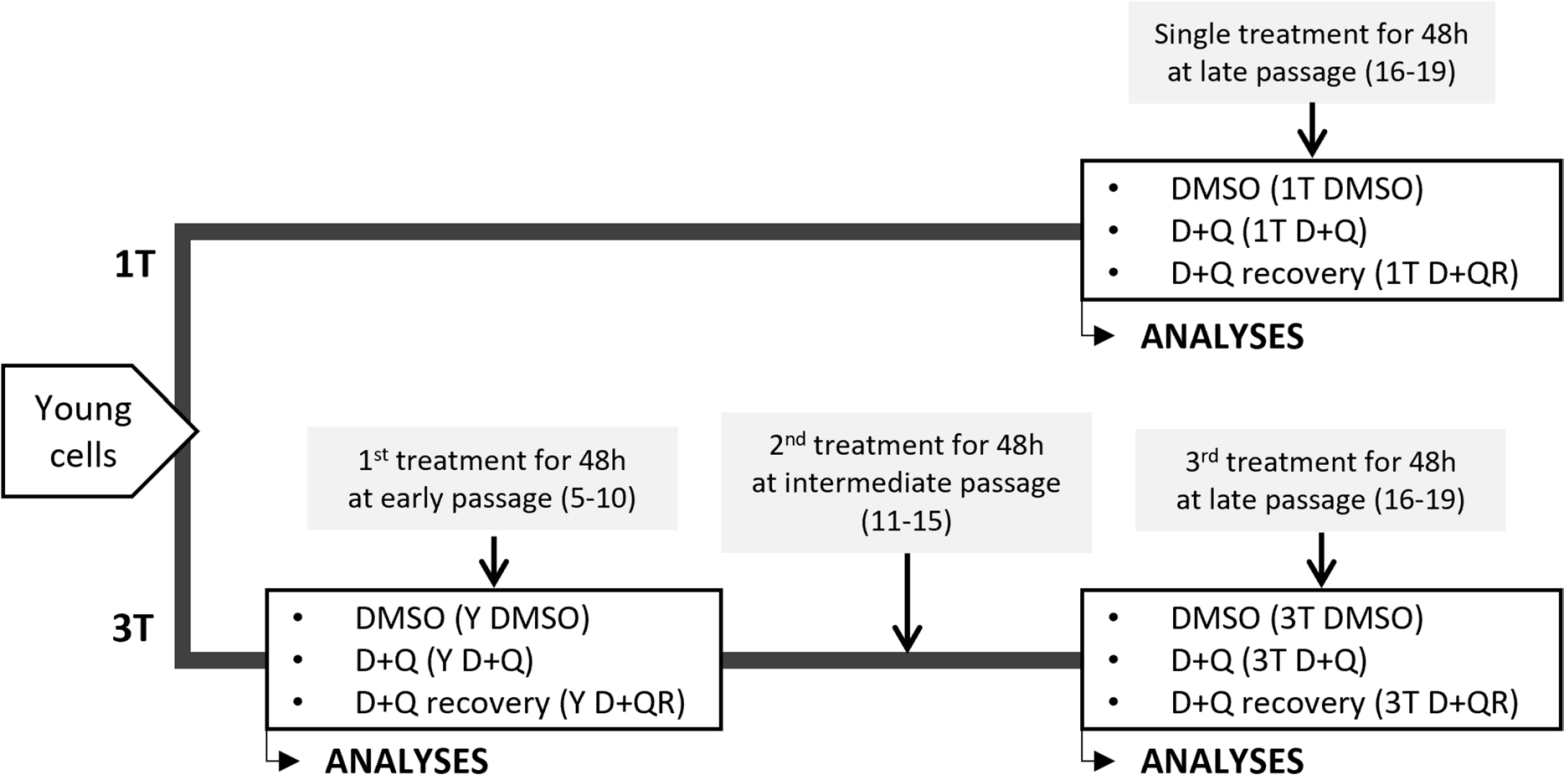
Experimental design with two types of treatment with D + Q. In the first variant, cells were treated one time with D + Q for 48 h at the late passage (passage 16–19) and analyzed directly after incubation with drugs or after 24 h of recovery in a drug-free medium. In the second approach, cells were treated with D + Q three times for 48 h, at the early (passage 5–10), middle (passage 11–15) and late passages (passage 16–19) and analyzed directly after 48 h treatment or after 24 h of recovery in a drug-free medium. Control cells were incubated for 48 h with DMSO at the same concentration as D + Q treated cells. **Y DMSO** – young control cells treated with DMSO for 48 h; **Y D + Q** – young cells treated with D + Q for 48 h; **Y D + QR** – young cells treated with D + Q for 48 h and allowed to recover for 24-h in the fresh medium; **1 T DMSO**–control late-passage cells treated once with DMSO for 48 h; **1 T D + Q** – late-passage cells one-time treated with D + Q for 48 h; **1 T D + QR** – late-passage cells one-time treated with D + Q for 48 h and allowed to recover for 24-h in a fresh medium; **3 T DMSO** – control late-passage cells triple-treated with DMSO for 48 h; **3 T D + Q** – late-passage cells triple-treated with D + Q for 48 h; **3 T D + QR** – RS cells triple-treated with D + Q for 48 h and allowed to recover for 24-h in fresh medium

One of the spectacular changes related to cellular senescence concerns the chromatin structure [13]. It has been observed that with senescence, chromatin becomes less condensed, the ratio of heterochromatin to euchromatin decreases, and constitutive heterochromatin (telomeric and centromeric regions) detaches from the nuclear lamina (e.g. due to a decreased expression and level of lamin B1). On the one hand, this affects gene expression and, on the other hand, it makes the loose DNA more susceptible to damage [14]. Our study aimed to analyze the impact of D + Q on the chromatin structure of young and senescent vascular smooth muscle cells (VSMCs). This cell type is directly involved in the development and progression of atherosclerosis and cardiovascular problems [15]. VSMCs were treated once or three times with the D + Q mixture at different stages of culture (the scheme of cell treatment is illustrated in Fig. 1). We analyzed the impact of D + Q on nuclear size and shape and chromatin pattern complexity (using CellProfiler software analysis) and on the course of replicative senescence (by analyzing the most common senescent markers related to nuclear alterations). The CellProfiler analysis showed that D + Q altered both young and senescent cells' nucleus morphology and chromatin pattern complexity but the direction of changes seemed different. Young cells took on the features of senescent ones, and senescent cells acquired features characteristic of young cells. The alterations in young cells appeared to be indicative of temporary senescence, with all changes reverting to their pre-treatment state. The results suggest that the triple treatment showed more beneficial effects on the nuclear morphology than a single one, and only after such treatment did some “rejuvenation” symptoms remain permanent in senescent cells. Considering senescence markers, the cells treated three times demonstrated more pronounced symptoms of senescence than cells treated only once. However, we cannot exclude that the effect was, at least partially, due to DMSO, as indicated by data obtained on control cells (1 T DMSO vs 3 T DMSO). Moreover, our data confirmed the previously observed difference in the sensitivity of various cell types to senolytics. VSMCs were the least sensitive, and preadipocytes were the most sensitive. Our results showed that the impact of senolytics concerns both young and senescent cells, and the final impact depends on the cell type and physiological context.

## Materials and methods

### Cell cultures

Human vascular smooth muscle cells (VSMC) derived from aorta (ATCC, Gibco) were cultured in Vascular Cell Basal Medium (ATCC) supplemented as defined by the manufacturer; human skin fibroblasts (ATCC, Gibco) were cultured in Dulbecco’s Modified Eagle’s medium (DMEM, Sigma-Aldrich/Merck) supplemented with 10% fetal bovine serum (FBS, BioWest), antibiotic and antimycotic mix solution (100 U/ml penicillin, 100 µg/ml streptomycin, 250 ng/ml amphotericin B, Sigma Aldrich); visceral preadipocytes (ScienCell) were cultured in Preadipocyte Medium (ScienCell) supplemented as defined by the manufacturer. All cell types were obtained from at least 3 young male donors aged 29–34 years. Cells were cultured in a humidified atmosphere with 5% CO_2_ at 37 °C. Cells were seeded in T75 culture flasks at 4,000 cells/cm^2^ density and passaged when they reached 70–80% confluency until they reached the state of replicative senescence. To recognize the cell population as senescent, it should contain at least 70% of SA-β-Gal-positive cells and no more than 30% of BrdU-positive cells.

### Experimental design

#### Impact of D + Q concentration on the proliferation of different types of normal cells

Cells (VSMC, fibroblasts, and preadipocytes) on early passages, also referred to as young cells, were cultured in 6-well plates and treated with dasatinib and quercetin (D + Q) 24 h after seeding. Dasatinib was added in 3 concentrations: 50 nM (D50), 100 nM (D100) and 200 nM (D200), while quercetin concentration remained constant at 5 µM, because earlier studies showed no impact even of high quercetin concentrations on cell proliferation and viability. Dasatinib is an anticancer drug that induces DNA damage and cell death in some concentrations. We have looked for concentrations that will be neither cytotoxic nor cytostatic. Control cells were treated with DMSO (solvent for D + Q). Cell proliferation was assessed by cell counting and using BrdU incorporation assay after 48 and 72 h of treatment.

#### Impact of the frequency of D + Q treatment on VSMC senescence and chromatin pattern complexity

VSMCs were cultured in T75 tissue flasks and treated with a D + Q mixture (100 nM and 5 µM, respectively) in two parallel experimental variants, differing in the frequency of treatment. The concentrations of D + Q in the mixture were not harmful to cells and did not show cytostatic or cytotoxic effects. First variant (1 T): cells were treated once on a late passage (passage 16–19) when at least 70% of cells were SA-β-Gal positive and did not proliferate. D + Q was added for 48 h. Cells were collected for analysis after 48 h with D + Q (1 T D + Q) or after a subsequent 24-h recovery in a fresh medium (1 T D + QR). Control cells were treated with DMSO for 48 h. Variant II (3 T): cells were treated 3 times with D + Q. The first treatment was given at an early passage (passage 5–10), the second in the middle passage (passage 11–15), and the third in the late passage, matching the single-treatment variant in the 1 T group (passage 16–19). Treatments were spaced to allow for 4 passages between each D + Q administration. Cells were treated with D + Q in the same manner as in the first variant and were collected similarly, including the recovery variant. Only early and late-passage-treated cells were taken for further analyses. Control cells were treated with DMSO for 48 h. The experimental design is visualized in Fig. 1.

### Senescence-associated β-Galactosidase activity

Cells were stained according to Dimri [16]. Cells were fixed (2% formaldehyde, 0.2% glutaraldehyde, PBS), washed in PBS and incubated overnight at 37 °C in the dark in staining buffer (5 mM potassium ferrocyanide, 5 mM potassium ferrycyanide, 150 mM NaCl, 2 mM MgCl2, 0.02 M phosphate buffer and X-Gal at concentration 1 mg/ml, pH 6.0.). Nuclei were stained with DAPI (1 µg/ml). Cells were analyzed using a light microscope (a Nikon Eclipse Ti fluorescence microscope). The results are expressed as % of cells with increased enzyme activity (blue colour) to all cells (based on DAPI-stained nuclei).

### Bromodeoxyuridine (BrdU) incorporation assay

Cell proliferation expressed as the ability to replicate DNA was assessed using the BrdU incorporation assay. Cells were seeded on 15 × 15 mm glass cover slips placed in a 12-well plate, and BrdU (BD Pharmigen) at 10 µM concentration was added to the medium. Cells were then incubated for 24 h. Next, cells were washed with PBS, fixed with ice-cold 70% ethanol and stored at −20 °C for at least 24 h. After thorough washing with 0.5% Triton X-100 (Sigma-Aldrich) solution in PBS and subsequent incubation with 2N HCl for 30 min at RT, cells were washed with PBS and incubated in 0.1 M sodium tetraborate decahydrate solution (Na₂B₄O₇·10H₂O, also known as borax) for 1 min at RT. Next, cells were immunostained with anti-BrdU primary antibody (Becton Dickinson, 1:100) dissolved in 1% bovine serum albumin (BSA; Biowest) in PBS containing 0.5% Tween-20 (Sigma-Aldrich) for 1.5 h at RT. After incubation, cells were washed with 0.5% Tween-20 in PBS and incubated with a secondary Alexa Fluor 488-conjugated antibody (Thermo Fisher Scientific, 1:500) dissolved in PBS for 1 h at RT. DNA was stained with DAPI (1 µg/ml) for 10 min at RT. The ratio of BrdU-positive cells to DAPI stained cells was calculated using ImageJ software.

### Immunofluorescence

Cells that were seeded on 15 × 15 mm glass cover slips placed in 12-well plates were fixed with 4% PFA for 15 min at RT, washed with PBS and stored in ethanol at −20 °C. Then, cells were permeabilized with 0.5% Triton X-100 solution in PBS for 10 min, blocked in blocking buffer consisting of 1.5% goat serum, 2% bovine serum albumin and 0.5% Triton X-100 in PBS for 10 min and incubated with primary antibody dissolved in blocking buffer for 2 h at RT. Subsequently, cells were washed in PBS and incubated with secondary antibody Alexa Fluor 488 or Alexa Fluor 555 dissolved in PBS (1:500, Thermo Fisher Scientific) for 1 h at RT. DNA was stained with DAPI (1 µg/ml) for 10 min at RT. Primary antibodies were used: 53BP1 (Novus Biologicals, #NB100-304, 1:500). Cells were analyzed using a fluorescent microscope Nikon Eclipse Ti.

### Western blot

Whole-cell protein lysates were prepared according to Laemmli [17]. After electrophoretic separation in Tris–glycine SDS running buffer (25 mM Tris–HCl, pH 8.3, 250 mM glycine, 0.1% SDS), proteins were transferred in the transfer buffer (25 mM Tris–HCl pH 8.3, 192 mM glycine, 0,5% SDS, 20% methanol) to a nitrocellulose membrane (Amersham GE Healthcare) which was blocked in 5% low-fat milk or BSA (in case of HP1α) in TBS containing 0.1% Tween-20 (TBST). Incubation with primary antibodies dissolved in blocking buffer (BSA in case of HP1α) was conducted overnight at 4 °C. Then, membranes were washed in TBST and incubated with horseradish peroxidase-conjugated secondary antibody (Dako, Agilent, 1:2000) diluted in low-fat milk, washed and visualized by enhanced chemiluminescence (ECL; Thermo Fisher Scientific). The signal was detected using X-ray films. Primary antibodies used: p53 (Santa Cruz, sc-126, 1:500), HMGB1 (Abcam, ab79823, 1:500), lamin B1 (Santa Cruz, sc-365962, 1:500), HP1α (Cell Signaling, #2616, 1:1000), H3K4me3 (Diagenode, C15410003, 1:1000), H3K9me3 (Diagenode, C15410193, 1:1000), H3K9Ac (Cell Signaling, #9649, 1:1000), H3K27me3 (Diagenode, C15410195, 1:1000), SiRT-6 (Cell Signaling, #12,486, 1:1000), GAPDH (Millipore, MAB374, 1:150000). Protein level was normalized to GAPDH and presented as a fold change relative to YDMSO. Hierarchical clustering was performed based solely on the GAPDH-normalized data using the Instant Clue tool [18].

### Nuclear morphology and chromatin pattern texture

The CellProfiler software was employed to analyze nuclear morphology and chromatin pattern texture. VSMCs cultured on 15 × 15 mm glass cover slides placed in 12-well plates were fixed with 4% PFA for 15 min and stained with Alexa Fluor 488 phalloidin (Invitrogen, 1:40) in PBS for 30 min at RT. DNA was stained with DAPI for 10 min. Imaging of the samples was performed using a wide-field DMI6000 (Leica Microsystems, Germany) fluorescence microscope equipped with a 40 × NA 0.5 objective (Leica Microsystems, Germany) and DCF 35DFXR2 camera (Leica Microsystems, Germany). Tile function in conjunction with software autofocus in LAS AF software (Leica Microsystems, Germany) was used to scan multiple single-plane fields of view covering most of each coverslip. Images were then exported to 8-bit TIFF format in FijiJ [19]. CellProfiler [20] and CellProfiler Analyst [21] were used to identify the nuclei and to measure the shape and texture parameters (Table 1). Texture measurements in the software were performed using the grey-level co-occurrence matrix (GLCM) [22] at a 3-pixel scale corresponding to ca. 850 nm. Texture parameters for all four dimensions were averaged for further analysis. To exclude out-of-focus objects, which constituted a significant part of all the nuclei, single-object images were assessed using the Microscope Focus Quality plugin in FijiJ [23]. Statistical analysis was performed in SAS® 9.4M7 software (SAS Institute Inc., USA): an equal number of in-focus objects were randomly chosen by the software from each of 3 biological experiment replicates and pooled. The data significantly deviated from a normal distribution; therefore, Kruskall-Wallis ANOVA followed by Dunn’s post-hoc test, was used to identify significant differences between the treatments. Custom SAS macro was used to perform the post-hoc test [24]. Differences were considered statistically significant at *p* < 0.05. For the clustering analysis (Fig. 6), the outlier measurements were discarded at the 1st and 99th percentile levels calculated individually for each parameter. Then, for each parameter, the values were rescaled to zero mean and unit variance. Finally, the median values for each of the parameter and treatment variants were clustered (default options—hierarchical algorithm with average linkage and Euclidean norm as distance function) and plotted using the *clustermap* function in the seaborn Python package.

**Table 1:**
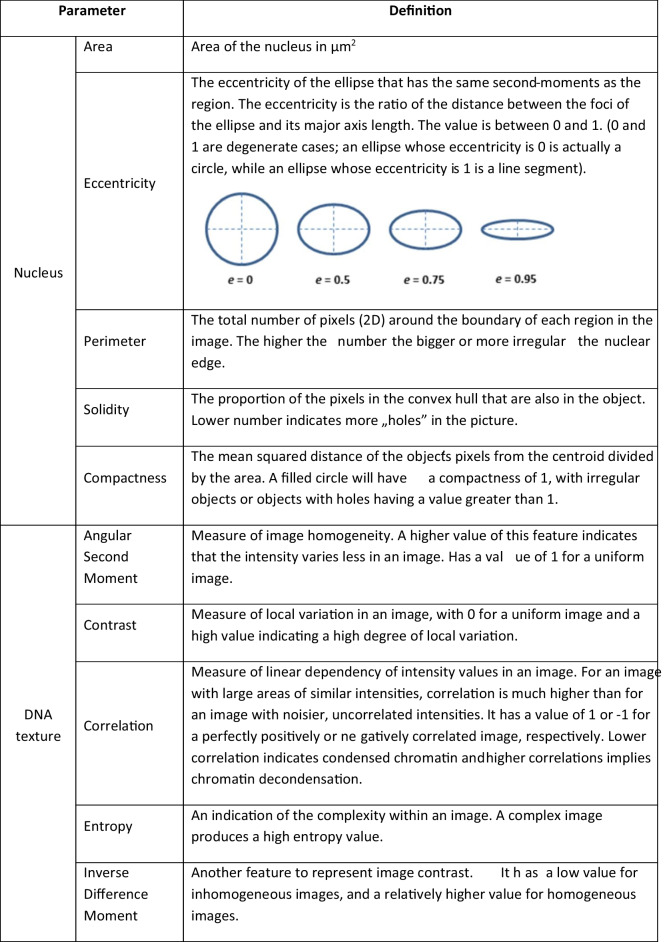
Summary of the parameters describing nuclear size and shape and DNA pattern texture. Taken from the software manual [https://cellprofiler-manual.s3.amazonaws.com/CellProfiler-3.0.0/modules/measurement.html]

### Statistics

**Fig. 2:**
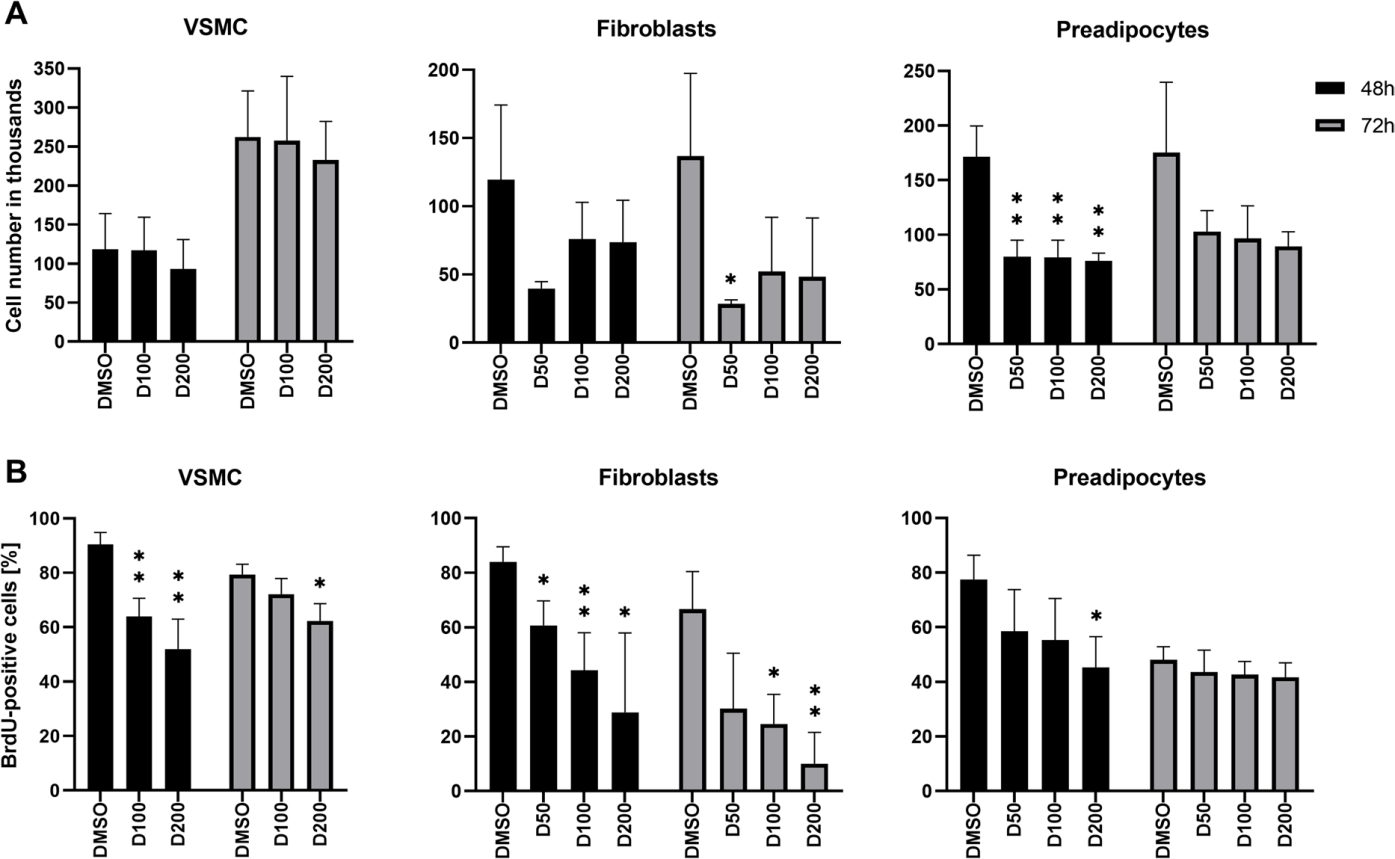
Sensitivity of VSMCs, fibroblasts and preadipocytes to D + Q treatment. Different concentrations of dasatinib (50, 100 and 200 nM for fibroblasts and preadipocytes, and 100 and 200 nM for VSMCs) were used with a constant quercetin concentration (5 µM) and the impact was analyzed after 48 and 72 h of treatment. **A** – total cell number, **B**—the percentage of BrdU-positives cells. N ≥ 3. A value of *p* < 0.05 was considered significant and statistical analysis was performed in relation to DMSO control within the group, i.e. 48 h and 72 h (*p < 0.05, ** *p* < 0.01, *** *p* < 0.001, **** *p* < 0.0001)

**Fig. 3:**
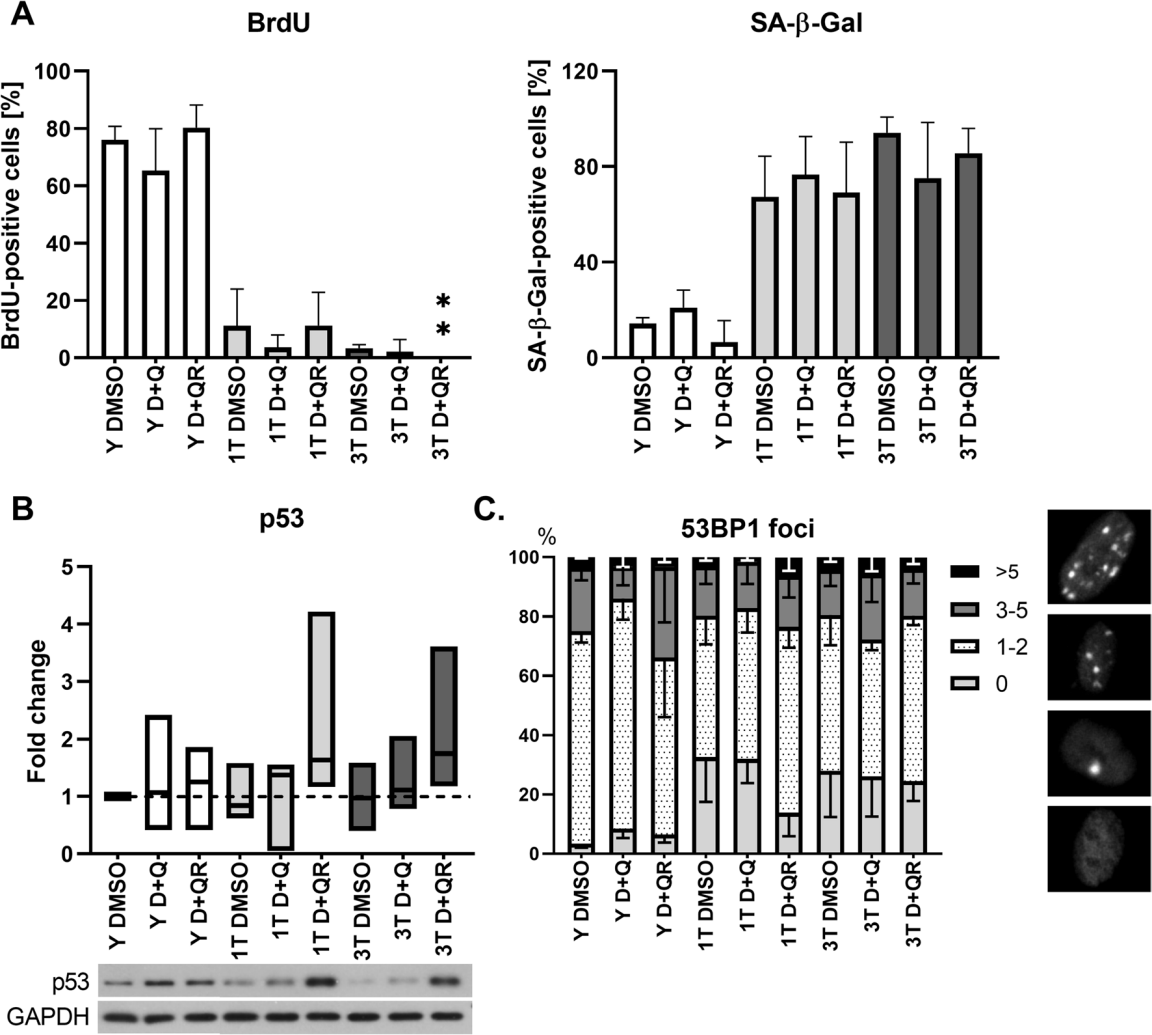
Impact of D + Q on proliferation, senescence and DNA damage in young and senescent VSMCs. Cells were treated in the late passage (1 T) or different passages (3 T). **A** BrdU-positive cells (left), and SA-β-Gal-positive cells (right), **B** densitometric evaluation of p53 protein analyzed by Western blotting. The middle line indicates the median, and the boxes represent the standard deviation. **C** DNA damage analyzed as a number of foci of 53BP1 protein: 0—without 53BP1 foci; 1–2—with 1 or 2 foci; 3–5—with foci number between 3 and 5; > 5—with more than 5 foci. The experiments were performed on cells derived from at least three donors, n > 3. Group description as in Fig. 1. Parametric two-tailed unpaired t-test. A value of *p* < 0.05 was considered significant (*p < 0.05, ** *p* < 0.01, *** *p* < 0.001, **** *p* < 0.0001)

**Fig. 4:**
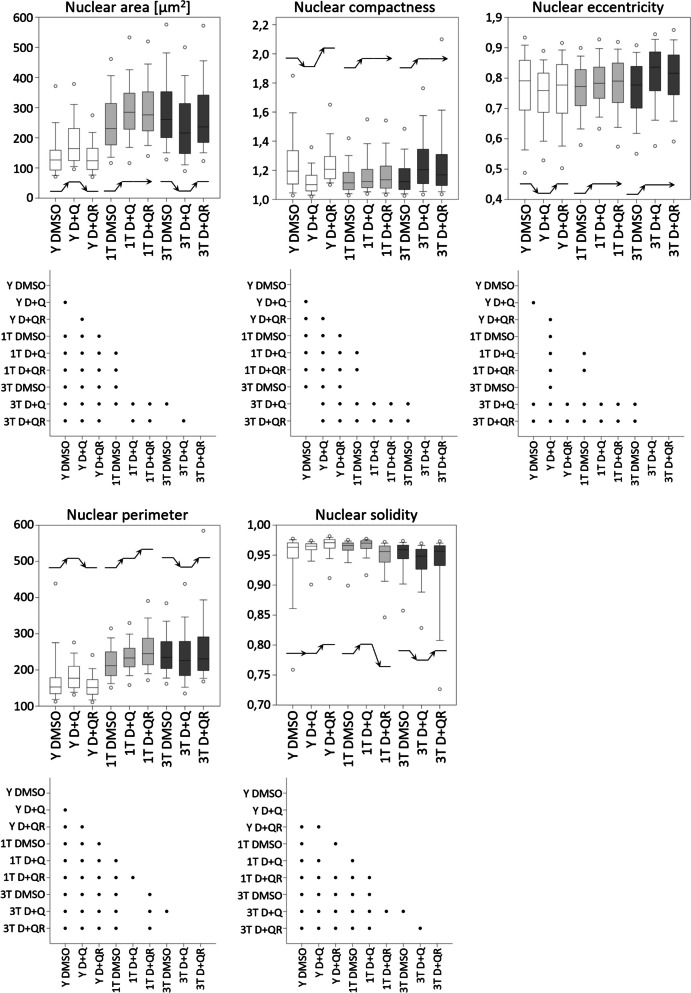
Selected parameters of nucleus characteristics: area, compactness, eccentricity, perimeter and solidity. Below each graphthere is a chart describing statistical significance. All parameters were described in detail in Table 1. The black lines ending with an arrowhead show the direction of changes after D + Q and recovery. *n* = 3, Kruskall-Wallis with Dunn's correction

**Fig. 5:**
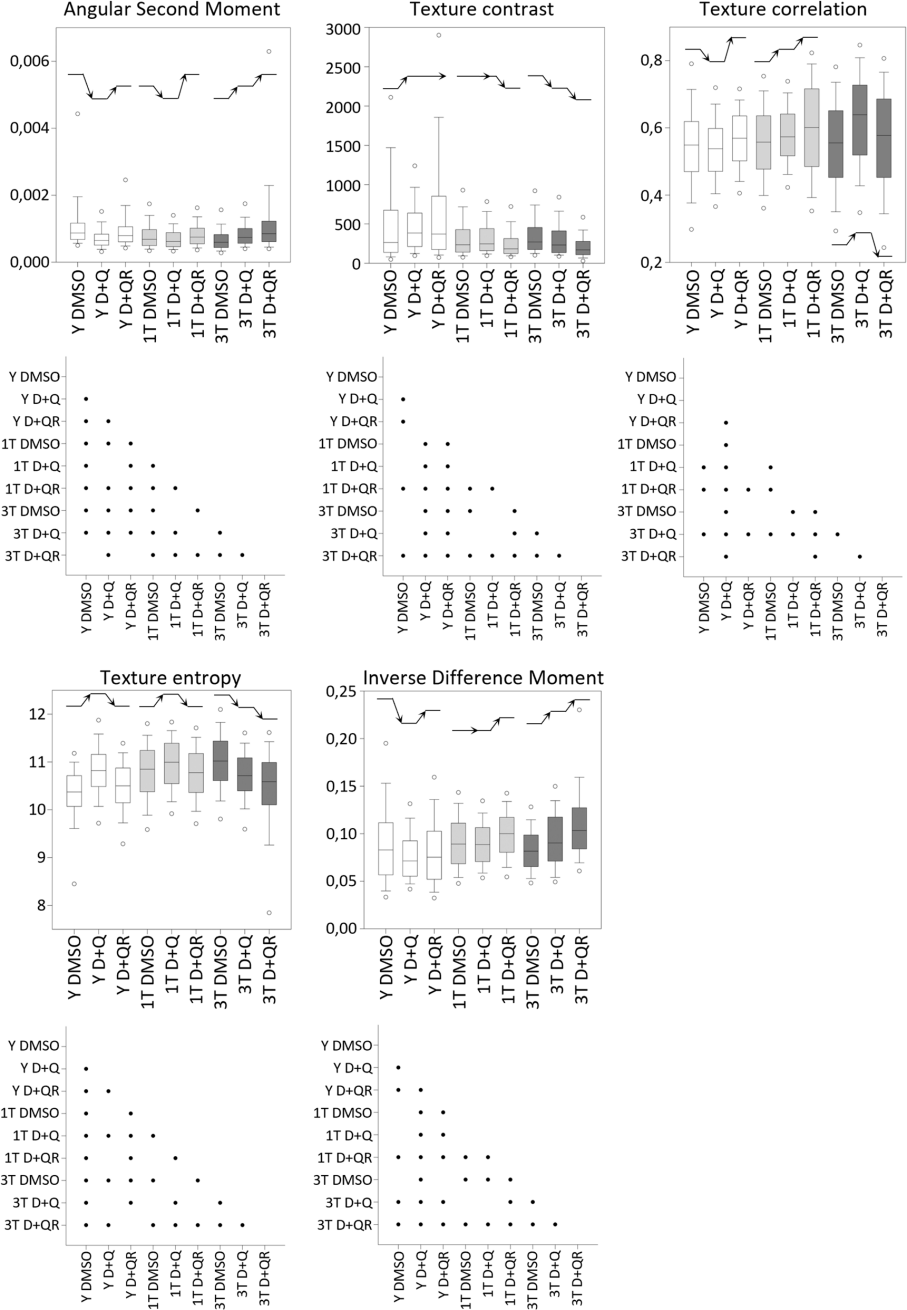
Selected parameters of DNA texture characteristics: angular second moment, contrast, correlation, entropy and inverse difference moment. Below each graph, there is a chart describing statistical significance. All parameters were described in detail in Table 1. The black lines ending with an arrowhead show the direction of changes after D + Q and recovery. *n* = 3, Kruskall-Wallis with Dunn's correction

**Fig. 6:**
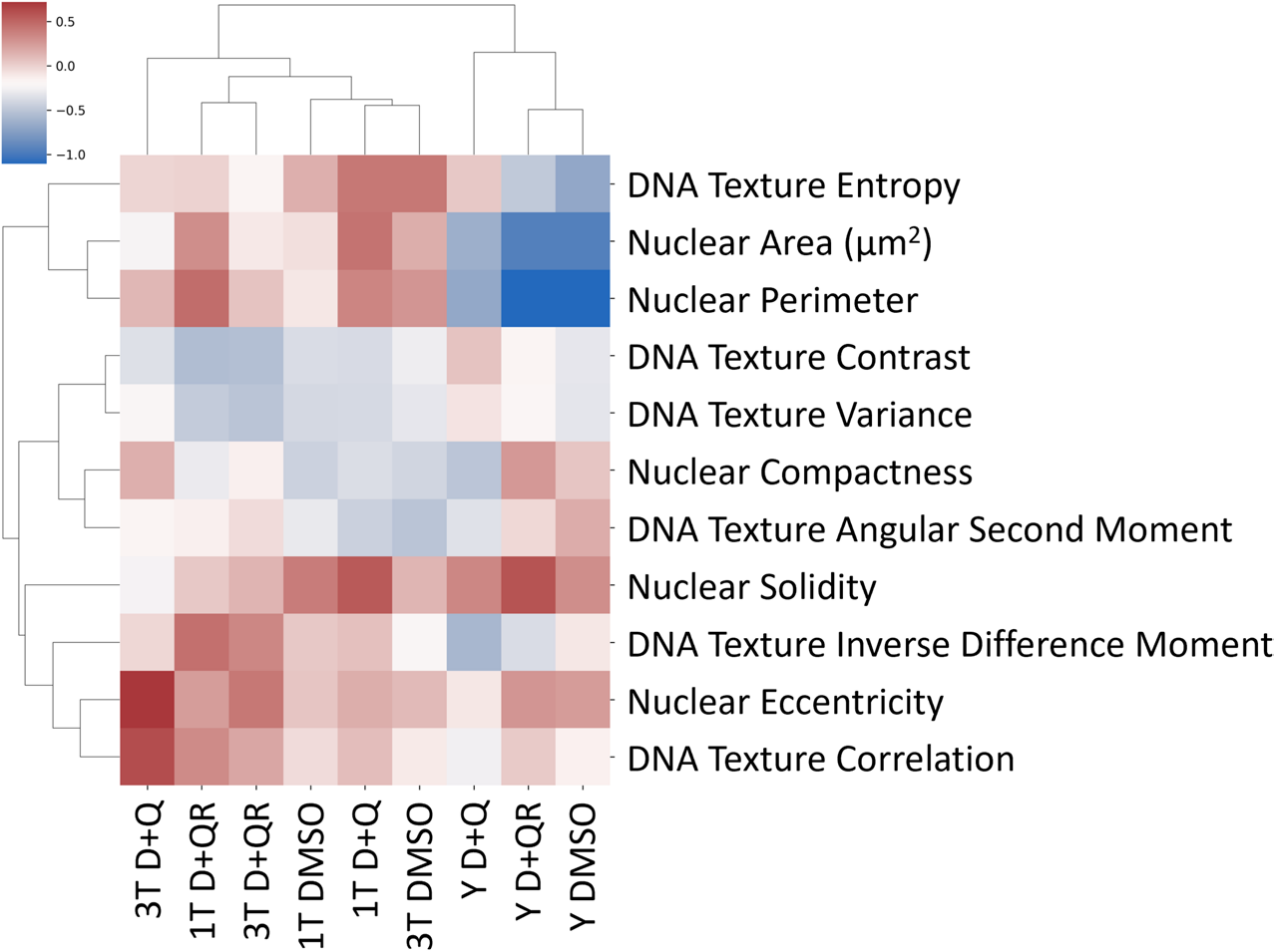
Summary of all nuclear and chromatin parameters presented in Figs. 5 and 6 plotted using the *clustermap* function in Python package

**Fig. 7:**
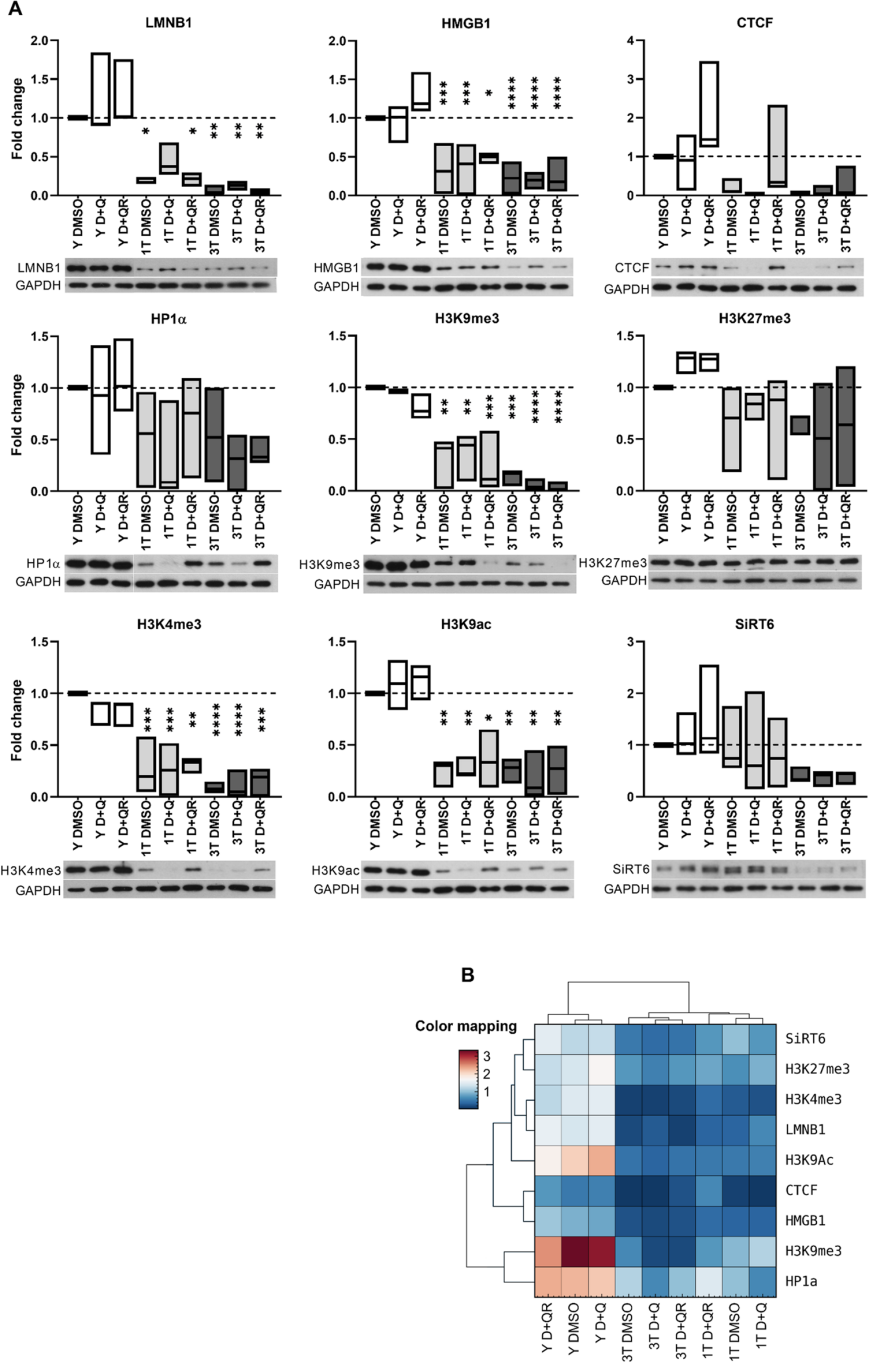
Expression profile of proteins related to senescence-associated chromatin alterations in young (Y) and senescent VSMCs treated with D + Q (1 T and 3 T). **A** Densitometric analysis of Western blot experiments performed on samples from 3 donors is presented as a fold change relative to Y DMSO (*n* = 3). Results are presented as boxplots where the line indicates the mean. The dashed line corresponds to the young cell control (Y DMSO). For statistical analysis, a one-way ANOVA followed by the Tukey's post-hoc test was performed to compare each experimental variant (*p < 0.05, ** *p* < 0.01, *** *p* < 0.001, **** *p* < 0.0001). On the graphs comparison to Y DMSO is shown while detailed statistical analysis is summarized in Supplementary Material (Sup.Tab.1). **B** Summary of protein expression profile relative to Y DMSO shown in the form of a heatmap. **C** Hierarchical clustering summarizing similarities between expression profiles of selected markers. The Color mapping scheme shows expression levels of proteins normalized to GAPDH

Statistical analysis was performed using GraphPad Prism (San Diego, California, USA) and SigmaPlot 12.3 (Systat Software). Analysis of sample distribution was performed using the Shapiro–Wilk test. The unpaired Student’s t-test or One-way ANOVA with Tukey’s post hoc test was applied for normally distributed samples, and for non-normally distributed samples—Kruskall-Wallis ANOVA followed by Dunn’s post-hoc test. Comparisons were performed to the control (DMSO) within the experimental group, i.e. within 48 h, 72 h, young, 1 T or 3 T variants (Fig. 2, 3 and 7A) or between each experimental condition (Fig. 4, 5, 6 and 7B). Unless stated otherwise, most graphs present mean ± SD from at least 3 independent experiments performed on cells from 3 different donors (*n* = 3). A value of *p* < 0.05 was considered significant (*p < 0.05, ** *p* < 0.01, *** *p* < 0.001, **** *p* < 0.0001). The following software was used to prepare the figures: ImageJ, GraphPad, Excel, clusterman in Python and Instant Clue.

## Results

### Sensitivity to D + Q treatment depends on cell type

To test cell sensitivity to D + Q treatment, three types of normal cells, VSMCs, fibroblasts and preadipocytes, were studied. Dasatinib toxicity was tested on young VSMCs at concentrations between 50 and 200 nM. Quercetin was used at a constant concentration (5 µM) that it is well-tolerated by most cell types as was shown by us (unpublished) and by others [4, 25]. VSMCs were less sensitive to D + Q treatment than fibroblasts and preadipocytes. The number of VSMCs after treatment with 100 and 200 nM concentration of dasatinib was similar to that of control cells (Fig. 2), but the number of BrdU-positive cells decreased after D + Q treatment (cytostatic, not cytotoxic effect). Fibroblasts were most sensitive to D + Q treatment. A decrease in the number of cells (suggesting cell death) and in the number of BrdU-positive cells was more pronounced than in the case of VSMCs. Preadipocytes responded stronger than VSMCs but weaker than fibroblasts; however, fibroblasts and preadipocytes were sensitive to DMSO (solvent for D + Q) since, although the number of cells after 48 and 72 h remained unchanged, the number of BrdU-positive cells decreased. Since the study aimed to check the long-lasting effect of D + Q on the chromatin structure, VSMCs were selected for further analysis as the least sensitive, and the concentrations 100 nM/5 µM (dasatinib/quercetin) were chosen for subsequent experiments.

### The impact of D + Q treatment on senescence and DNA damage of VSMCs

To establish the impact of D + Q (100 µM/5 µM, respectively) on the VSMC senescence rate, selected senescence markers were analyzed in young and senescent cells (experimental approach as in Fig. 1). We have studied the ability to replicate DNA, SA-β-Gal activity (Fig. 3A, Fig. S1), p53 level (Fig. 3B, Fig. S2) and the level of DNA damage (detection of 53BP1 foci, Fig. 3C). In the population of cells treated with single dose (1 T), slightly more BrdU-positive and fewer SA-β-Gal-positive cells were observed; however, the results are not statistically significant. It was also observed that repeated treatment with DMSO might have had a slight influence on SA-β-Gal activity as the difference between 1 T DMSO and 3 T DMSO treated cells, albeit statistically insignificant, neared 30% (Fig. 3A). The percentage of SA-β-Gal-positive cells in the population of control cells was: Y DMSO 14%, 1 T DMSO 67%, 3 T DMSO 94%. D + Q treatment did not impact DNA damage in VSMCs, analyzed as a number of 53BP1 foci (a marker of DNA; DSB—DNA double-strand brakes). To quantify DNA DSB cells were categorized into four groups: without 53BP1 foci; with 1 or 2 foci; with 3–5 foci; and with more than 5 foci. No significant differences were observed between treatment types (1 T vs 3 T). The amount of p53 protein did not change in young cells but seemed to be elevated in senescent D + Q-treated cells (1 T and 3 T); however, only in the recovery variant (D + QR). This can be a result of cell response to stress. Treatment with D + Q of both young (Y D + Q) and senescent (1 T D + Q) cells impacts cellular senescence only slightly and temporarily (increased number of SA-β-Gal-positive and decreased BrdU-positive cells). In 3 T D + Q and 3 T D + QR cells, proliferation was inhibited. 3 T cells seemed more advanced in cellular senescence than 1 T cell (Fig. 3A). However, this was only a tendency considering the lack of statistically significant differences. We can conclude that D + Q treatment only slightly impacted proliferation and senescence pace (observation of cells in culture did not show a faster rate of senescence for cells treated three times) and did not induce DNA damage in either young or senescent cells.

### D + Q mixture affects chromatin structure differently in young and senescent VSMCs

To analyze the long-lasting impact of D + Q treatment on nucleus architecture and chromatin structure, we used CellProfiler. On the one hand, senescence-associated chromatin alterations are strongly related to global chromatin relaxation and, on the other hand, to local chromatin condensation, both of which lead to changes in gene expression [13]. VSMCs were treated with a D + Q mixture, as shown in Fig. 1. The nuclei were stained with DAPI and scanned with the microscope. We checked the selected parameters of the nucleus and DNA staining texture (as detailed in Table 1).

We have analyzed various parameters of the nucleus, such as area, solidity, perimeter, eccentricity, and compactness (Fig. 4), and of DNA staining texture such as inverse difference moment, entropy, correlation, contrast and angular second moment (Fig. 5). Our analysis allowed us to identify features that were common to both young and senescent cells (Y DMSO vs. 1 T DMSO and 3 T DMSO). In general, senescent cells were characterized by larger and more regular nuclei than young cells. This was reflected by a bigger nucleus area and longer perimeter, and by lower eccentricity and compactness values. Moreover, cell senescence was accompanied by a more complex, less uniform DNA staining pattern as revealed by texture parameters: higher entropy and lower angular second moment. These changes were observed in both 1 T and 3 T cells; however, they were more prominent in 3 T cells for most parameters: area, perimeter, entropy and angular second moment. Alterations in nuclear shape solidity were inconclusive. In this analysis, no impact of periodical treatment with DMSO was detected.

We analyzed the impact of the D + Q mixture on young and senescent cells. In young cells, the D + Q mixture caused some changes toward the senescent phenotype: bigger nuclear area, more regular nuclear shape (lower eccentricity and higher compactness), along with higher complexity of the DNA staining pattern reflected by higher entropy, lower angular second moment and lower inverse difference moment. However, most of those changes were temporary and reverted to the initial state after 24 h in the fresh medium (Figs. 4 and 5). Similarly to the situation in young cells, in 1 T cells, some of the studied parameters, such as: higher nuclear area, longer perimeter, higher eccentricity, entropy and lower angular second moment, also changed toward the senescent phenotype after treatment. In the case of these cells, the size and shape parameters changed permanently, whereas changes in texture parameters were temporary. It should be noted that the nuclei of 3 T cells exhibited a more juvenile phenotype after D + Q treatment, characterized by: smaller nuclear area and perimeter, more elongated and less regular shape, and more uniform DNA staining pattern (lower entropy, higher angular second moment and inverse different moment). Changes to the nuclear size were revertible in this case; however, the shape of the nucleus and the chromatin staining texture were changed permanently.

To conclude, we have shown that D + Q affected the chromatin of both young and senescent cells; however, the direction of changes was different and also depended on the number of treatments. Young cells and senescent cells treated with D + Q only once resembled senescent cells in several characteristics. On the other hand, senescent cells subjected to repeated treatment showed some signs of “rejuvenation”. It must be noticed that changes in all parameters were relatively subtle and some disappeared after the recovery. Compared with young control cells, the most different variant was 3 T D + Q (Fig. 6).

### Impact of single and triple D + Q treatment on selected proteins recognized as markers of senescence-associated chromatin alterations

We analyzed the senescence markers in VSMCs. These include lamin B1, HMGB1, CTCF, HP1α, H3K9me3 (histone H3 trimethylated at lysine 9), H3K27me3 (histone H3 trimethylated at lysine 27), H3K4me3 (histone H3 trimethylated at lysine 4), H3K9Ac (histone H3 acetylated at lysine 9) and Sirt6. Lamin B1 builds the nuclear lamina and is responsible for proper nucleus architecture and anchoring specific chromatin regions to the nuclear envelope. HMGB1 binds to DNA and changes the spatial structure of gene promoter sites to facilitate the access of transcription machinery and regulate gene expression. CTCF is a DNA-binding protein involved in the multi-level organization of the genome. HP1α, H3K9me3, and H3K27me3 are heterochromatin markers, and H3K4me3 and H3K9Ac are euchromatin markers. Sirt6 is involved in DNA repair, heterochromatin regulation, and genome and epigenome stability. The levels of the above-mentioned proteins drop in senescent cells [14, 26]. The proteins were analysed by WB. As presented in Fig. 7, all these proteins decreased in senescent VSMCs.

D + Q treatment of young cells mainly caused elevation of the levels of the studied proteins. After recovery the levels were similar or even higher comparing to Y DMSO. The exceptions were H3K9me3 and H3K4me3, whose levels decreased after D + Q treatment and did not reach the basic level after recovery. In senescent cells, the effect of D + Q treatment was very diverse and depended on the protein and experimental variant. The most pronounced decrease in all proteins was observed in 3 T cells (Fig. 7). It has to be highlighted that multiple treatments, even with DMSO, were not neutral for cultured cells. This could be concluded by comparing 1 T DMSO with 3 T DMSO treated cells (diminished levels of LMNB1, HMGB1, CTCF, H3K9me3, H3K4me3, Sirt6). However, we can only talk about tendency because the observed changes were not statistically significant (Table 1 in Supplementary Material). This may suggest that the 3 T DMSO cells had more strongly pronounced senescence markers due to exposure to DMSO rather than repeated exposure to D + Q.

The results showed apparent differences between young and senescent cells. They also showed differences between senescent cells depending on the number of treatments (1 T and 3 T). In 3 T cells, changes in the level of the studied proteins during senescence were similar in the D + Q and D + QR variants; however, in 1 T cells, the D + QR variant deviated from D + Q, which suggests that cells resembled some features typical for young cells. In Fig. 7B, we summarized the protein expression levels normalized to Y DMSO. Additionally, we performed hierarchical clustering of all proteins normalized to GAPDH level. This analysis aimed to identify similarities in protein expression patterns across different experimental conditions as well as between variants. The results revealed two distinct groups of cells: young and senescent. Furthermore, differences between the 1 T and 3 T conditions were evident. In the 3 T group, D + Q and D + QR treated cells exhibited similar protein expression patterns, differing significantly from cells treated with DMSO. This suggests that protein expression does not revert to the pre-treatment state, indicating permanent changes. In the 1 T group, DMSO and D + Q treated cells showed similar patterns, while the D + QR variant was the most divergent. When it comes to changes in protein levels those of H3K9me3 and HP1α were the most similar and, at the same time, most divergent from others. Other protein pairs undergoing similar quantitative changes were HMGB1 and CTCF, H3K4me3 and LMNB1, SIRT6 and H3K27me3.

Summarizing, the levels of all tested proteins decreased in senescent cells (S vs Y). Considering the proteins recognized as senescence markers, the D + Q treatment was more prosenescent for 3 T cells. This contradicts the CellProfiler analysis, which pointed to the 3 T treatment variant as the one resembling young cells. However, contrary to the CellProfiler analysis, where DMSO did not appear to affect nuclear morphology, in the protein analysis the impact of treatment with DMSO could not be neglected because control cells treated periodically with the solvent had more pronounced senescent markers than cells treated only one time.

## Discussion

In 2015 it was shown that the D + Q mixture had a selective effect on senescent cells [4]. The efficacy of D + Q has been confirmed for both cell treatment and administration in animals [3, 4]. Old and atherosclerotic mice treated long-term with the D + Q mixture showed less vasomotor dysfunction than untreated mice [27]. Mice treated once a month with the D + Q mixture for 4 months showed a reduced number of senescent osteocytes [28]. However, a single treatment of naturally aged mice also effectively reduced senescence markers in fat tissue cells and improved cardiovascular system functioning [4]. When accelerated senescence was induced by limb irradiation in mice, administering a single dose of D + Q improved exercise capacity for a few months [4]. It was shown that even the treatment of old animals resulted in beneficial effects (prolonged life and better condition) [27, 29, 30].

Since D + Q has been subjected to clinical trials in some aging-related diseases and is also considered for use in a prophylactic approach it is essential to study the impact of D + Q treatment on chromatin structure, especially in young cells. Till now, several clinical trials have been started, and at least three have been completed [31–33; https://clinicaltrials.gov]. One of the diseases in which the usefulness of the mixture was tested is diabetic kidney disease (DKD) [9]. It is reasonable to target aging per se, which can delay age-related alterations. The long-term impact on young cells must be known to eliminate the risk of undesirable side effects.

Usually, senolytics are applied to eliminate senescent cells because their mechanism of action is to sensitize cells to cell death. In other studies, the doses of D + Q used were between 5–50 µM and 0.5–1.5 nM, respectively. Such doses reduced some symptoms of senescence in fibroblasts, neurons or oligodendrocyte progenitor cells [25, 34, 35]. In studies by Zhu and coworkers, the concentration of quercetin in the mixture was 20 µM, and different dasatinib (50–800 nM) concentrations were tested [35]. The HUVECs were more sensitive to D + Q than preadipocytes. Our study intended to analyze how the treatment impacted young cells, especially the chromatin structure and nucleus architecture. Therefore, we have used lower doses of these drugs. We also wanted to establish the effectiveness of a single or multiple treatment. Since changes in the nuclear architecture and chromatin structure related to aging entail significant consequences for cell functioning, understanding the mechanism of action of D + Q on the cell nucleus will allow us to estimate the safety and effectiveness of senotherapy. Furthermore, such a study is essential because a phase I pilot study concerning the impact of D + Q on DNA methylation clocks has already been performed. The results suggested a significant increase in epigenetic age acceleration (EAA) when estimated by the Horvath pan tissue and Hannum EAA clocks (first generation epigenetic clocks) but no effects were detected according to the second and third generation of epigenetic clocks [36].

Until now, there have been no data showing the impact of D + Q on chromatin structure and appearance of senescence markers in young cells, and data showing long-term effects of D + Q on young cells are scarce. Our studies show that in young cells, D + Q causes a shift towards senescence characteristics (senescence markers, nucleus and DNA texture alterations). However, this effect is transient and disappears after 24 h of recovery in a fresh medium as evidenced by most of the analyzed indicators, i.e. a temporary decrease in BrdU-positive cells and an increase in SA-β-Gal-positive cells. Furthermore, this is not fully supported by results concerning recognized senescence markers, the level of which changes slightly (no statistical significance), and the direction of changes is not coherent. SA-β-Gal activity increases temporarily in 1 T senescent and young cells treated with D + Q, probably due to stress. Considering the SA-β-Gal, multiple treatments seem more senescence-protective (fewer SA-β-Gal-positive cells in 3 T D + Q, and in 3 T D + QR than in 3 T DMSO) than single treatments (increased number of SA-β-Gal-positive in 1 T D + Q and similar in 1 T DMSO and 1 T D + QR). It can be concluded that multiple treatments, even at low doses, reduced senescent cell numbers immediately after the first and second treatments. On the other hand, analysis of proteins recognized as senescence markers (downregulated in senescent cells) (Fig. 7) showed a noticeable decline in all of them in 3 T, suggesting that treatment of cells at earlier passages was not neutral, and it can be interpreted that 3 T was less beneficial than 1 T. This was just a trend (lack of statistical significance) but observed consistently for all proteins. It has to be highlighted that, when it comes to senescence markers, multiple treatments, even with DMSO, were not neutral for cultured cells (1 T DMSO vs 3 T DMSO). Such a trend, suggesting a favourable effect of 1 T treatment, was not observed in CellProfiler analyses. The nucleus morphology suggested that multiple treatments reduced the symptoms of senescence to a greater extent than single treatments. Features resembling those of young cells appeared only when cells were treated repeatedly. Most literature data confirm a beneficial effect of the D + Q mixture on senescent cells as a result of both repeated cell treatment and administration to animals (analysis of animal condition and selected ex vivo cell senescence markers). Based on CellProfiler, we have observed a sort of temporary convergence in the characteristics of young and senescent cells as a result of D + Q treatment. However, it can be concluded that 3 T makes the chromatin of senescent cells more similar to that of young ones. Considering the nuclear area, one of the most spectacular and distinguishable parameters describing the nucleus, it can be observed that in young cells, the area transiently increases (becomes similar to the senescent ones), and in senescent 1 T cells, the increase in nuclear area is stable. The nucleus area of 3 T senescent cells is similar to that of young cells. This would suggest that the 3 T treatment variant gives better effects on cell rejuvenation. The transient increase in the nucleus area of young cells is probably due to the response to stress conditions caused by dasatinib, but it does not seem to be related to DNA damage, as D + Q treatment did not cause DNA DSB either in young or senescent cells. D + Q treatment mobilizes stress-responsive proteins (p53, p21), which may result in better protection of cells against stress.

The presented results confirmed that there is an increase in chromatin relaxation (increase in the surface of nuclei/uneven texture) during senescence. Based on the results regarding DNA texture parameters, it can be concluded that the nuclei of senescent cells have a more complex, heterogeneous staining pattern of DNA than those of young cells. Treatment with D + Q caused a significant change in the shape and surface of the nuclei, as well as in the texture parameters of the DNA staining pattern both in young or senescent cells. However, young and senescent cells responded differently to the treatment and recovery, and treatment with D + Q impacted 1 T and 3 T cells differently. In 3 T cells, the treatment caused the DNA pattern to become more uniform and less complex, and an opposite trend was observed in 1 T cells. Moreover, the nuclear areas of 1 T and 3 T cells had a similar staining intensity, distinct from that of young cells. Treating young cells with the mixture seems to affect chromatin compactness (increased nuclear area and perimeter and changes in DNA texture – the nuclear shape becomes more irregular and less elongated), making young cells more similar to senescent ones. However, the observed effect was only temporary/reversible. In the case of 1 T and 3 T senescent cells, the nuclei had a more regular and elongated shape, which made them look more similar to the nuclei of young cells, and this effect persisted despite recovery. However, the effect of “rejuvenation” was observed only after repeated treatment (3 T). In 1 T cells, the nuclei had a larger surface area after treatment, and this effect was irreversible. In the case of 3 T cells, the surface area decreases, but this was a reversible process. After recovery, most of the texture parameters returned to the control level for young (suggesting no harmful effect) and 1 T cells (suggesting lack of a “rejuvenation” effect), but for 3 T cells the change was permanent (suggesting some symptoms typical for young cells).

Assessment of the nuclear structure is a useful tool to recognize senescent cells in culture and estimate their overall condition [37]. Filippi-Chiela et al. have shown that young, senescent, mitotic and apoptotic cancer cells can be recognized in the cell culture based on the size and structure of their nuclei. It was proved that both the cells and their nuclei enlarge during senescence; however, after the D + Q treatment the nuclear shape of senescent cells resembled that of young control. Unfortunately, no information was provided in the context of chromatin texture.

## Conclusions

Our study confirmed the specificity of senolytics towards different cell types. The obtained results allow us to believe that the D + Q mixture could potentially affect cellular senescence, reversing its effects or alleviating its symptoms at the level of chromatin structure. Importantly, it has been shown that this effect is not unambiguous with regard to the commonly assessed senescence markers and that the mechanism of cellular senescence is complex and multidirectional. We have shown an effect of the D + Q mixture on the chromatin structure of both young and senescent cells. However, our results suggest that D + Q affects the chromatin structure differently in young and senescent cells, inducing an opposite response. After D + Q treatment, the chromatin structure of young cells acquires some features typical for senescent ones. The chromatin of senescent cells treated by D + Q resembles the chromatin structure in young cells to some extent. It is possible that the effect on chromatin-associated proteins and modifications is vital for the potential “rejuvenating” effect of the mixture. Chromatin alterations caused by D + Q in early passages (young, proliferating cells) seem transient. When we analyzed the senescence markers, the effect of D + Q on 1 T cells seemed to be more noticeable than on 3 T ones. Cells treated several times have the most pronounced senescent markers. However, observations obtained using Cell Profiler suggested that the chromatin and nucleus structure parameters of 3 T cells showed more signs of subtle resembling young cells effect. The results suggest that the beneficial effect of the D + Q mixture may be the result of not only the elimination of senescent cells but also of the “rejuvenation” of their chromatin and alterations in gene expression, including genes encoding proteins responsible for SASP.

## Supplementary Information

Below is the link to the electronic supplementary material.

Supplementary file1 (Fig. S1) Impact of D+Q on SA-β-Gal activity in young and senescent VSMCs. Representative images. SA-β-Gal-positive cells - blue color in the light microscope (upper panels), all cells were marked by nuclei staining (DAPI) – blue color in fluorescent microscope. Scale = 50 µm (PNG 10094 KB)

Supplementary file2 (Fig. S2) Impact of D+Q on DNA damage in young and senescent VSMCs. Representative images of DNA damage analyzed as a number of foci of 53BP1 protein. Green color – 53BP1, blue color – DNA/nuclei (DAPI staining) to visualized all cells. Scale = 50 µm (PNG 4936 KB)

Supplementary file3 (Sup.Tab.1) Detailed statistical analysis for data presented on Fig. 7A. Densitometric analysis of Western blot experiments performed on samples from 3 donors is presented as a fold change relative to YDMSO (n = 3). For statistical analysis, a one-way ANOVA followed by the Tukey's post-hoc test was performed to compare each experimental variant (* p < 0.05, ** p < 0.01, *** p < 0.001, **** p < 0.0001) (XLSX 13 KB)

## Data Availability

The datasets generated during and/or analyzed during the current study are available from the corresponding author on reasonable request.
