# Supplementary figures and images for: The senolytic cocktail, dasatinib and quercetin, impacts the chromatin structure of both young and senescent vascular smooth muscle cells
Source: GeroScience. 2025 Jan 20;47(3):3907–25. doi: 10.1007/s11357-024-01504-6 (PMC12181558; doi:10.1007/s11357-024-01504-6)

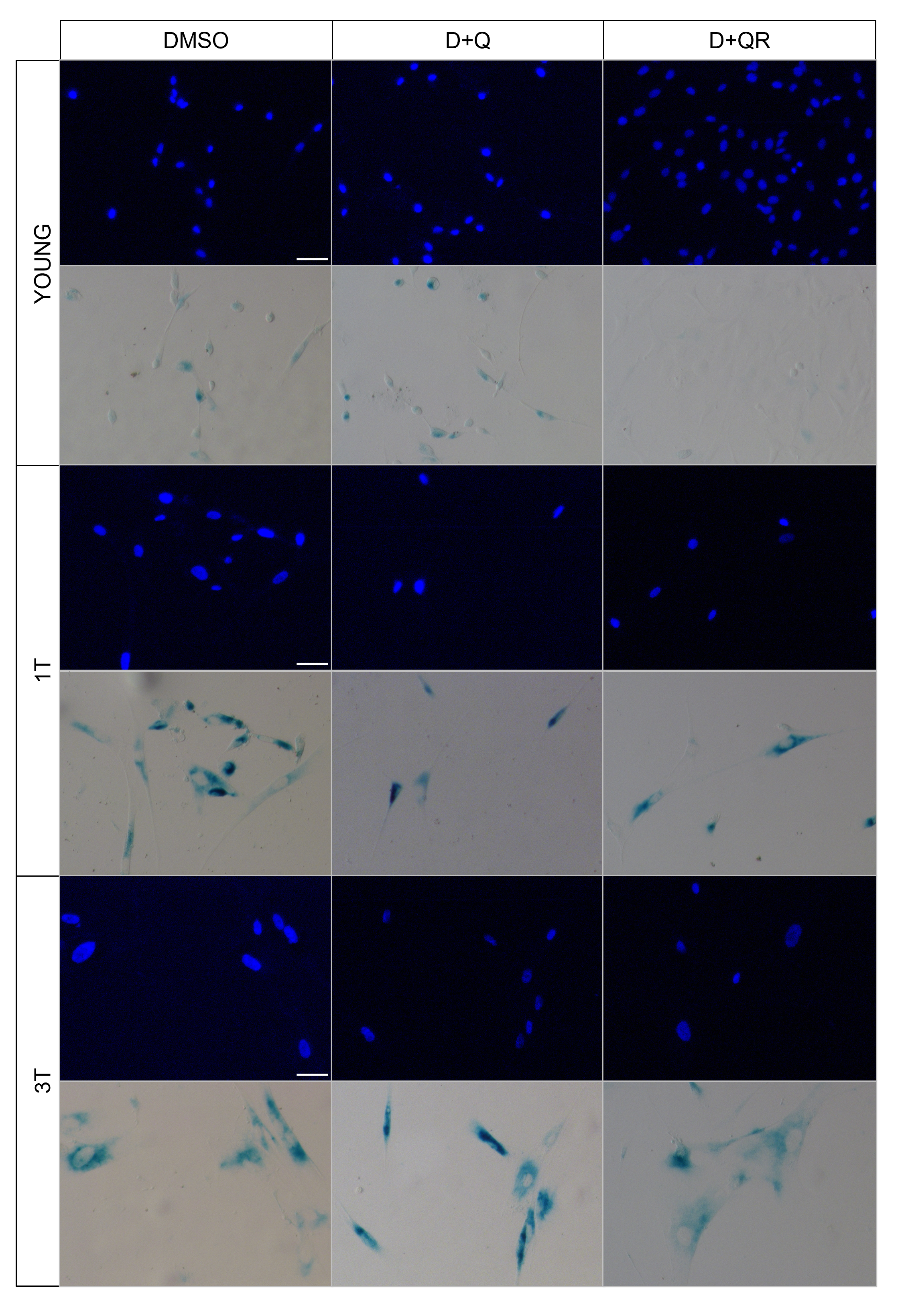

Supplement: Supplementary file 1 — Supplementary file1 (Fig. S1) Impact of D+Q on SA-β-Gal activity in young and senescent VSMCs. Representative images. SA-β-Gal-positive cells - blue color in the light microscope (upper panels), all cells were marked by nuclei staining (DAPI) – blue color in fluorescent microscope. Scale = 50 µm (PNG 10094 KB) [file 11357_2024_1504_MOESM1_ESM.png]

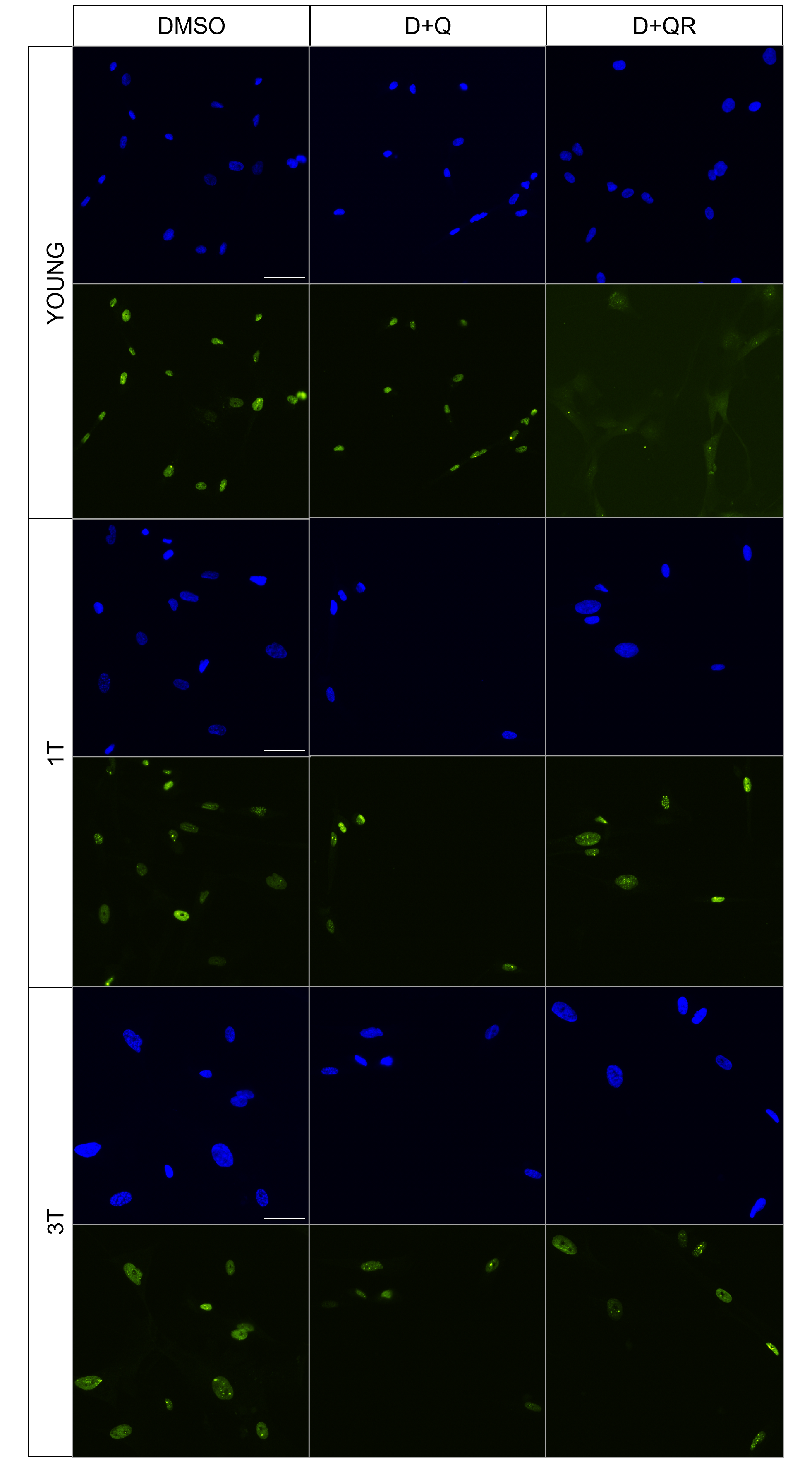

Supplement: Supplementary file 2 — Supplementary file2 (Fig. S2) Impact of D+Q on DNA damage in young and senescent VSMCs. Representative images of DNA damage analyzed as a number of foci of 53BP1 protein. Green color – 53BP1, blue color – DNA/nuclei (DAPI staining) to visualized all cells. Scale = 50 µm (PNG 4936 KB) [file 11357_2024_1504_MOESM2_ESM.png]
